# The Diagnostic Value of Gut Microbiota Analysis for Post-Stroke Sleep Disorders

**DOI:** 10.3390/diagnostics13182970

**Published:** 2023-09-17

**Authors:** Huijia Xie, Jiaxin Chen, Qionglei Chen, Yiting Zhao, Jiaming Liu, Jing Sun, Xuezhen Hu

**Affiliations:** 1 Department of Geriatrics, The Second Affiliated Hospital and Yuying Children’s Hospital of Wenzhou Medical University, Wenzhou 325027, China; xiehuijia163@163.com (H.X.); rm120w@163.com (J.C.); stepbystepchen44@163.com (Q.C.); 15867160109@163.com (Y.Z.); 2 Department of Preventive Medicine, School of Public Health and Management, Wenzhou Medical University, Wenzhou 325035, China; wzjiaming_liu@163.com; 3 Department of Emergency Medicine, The Second Affiliated Hospital and Yuying Children’s Hospital of Wenzhou Medical University, Wenzhou 325027, China

**Keywords:** sleep disorders, gut microbiota, acute ischemic stroke, prediction, biomarkers

## Abstract

Background: Gut microbiota have been associated with many psychiatric disorders. However, the changes in the composition of gut microbiota in patients with post-stroke sleep disorders (PSSDs) remain unclear. Here, we determined the gut microbial signature of PSSD patients. Methods: Fecal samples of 205 patients with ischemic stroke were collected within 24 h of admission and were further analyzed using 16 s RNA gene sequencing followed by bioinformatic analysis. The diversity, community composition, and differential microbes of gut microbiota were assessed. The outcome of sleep disorders was determined by the Pittsburgh Sleep Quality Index (PSQI) at 3 months after admission. The diagnostic performance of microbial characteristics in predicting PSSDs was assessed by receiver operating characteristic (ROC) curves. Results: Our results showed that the composition and structure of microbiota in patients with PSSDs were different from those without sleep disorders (PSNSDs). Moreover, the linear discriminant analysis effect size (LEfSe) showed significant differences in gut-associated bacteria, such as species of *Streptococcus*, *Granulicatella*, *Dielma*, *Blautia*, *Paeniclostridium,* and *Sutterella*. We further managed to identify the optimal microbiota signature and revealed that the predictive model with eight operational-taxonomic-unit-based biomarkers achieved a high accuracy in PSSD prediction (AUC = 0.768). *Blautia* and *Streptococcus* were considered to be the key microbiome signatures for patients with PSSD. Conclusions: These findings indicated that a specific gut microbial signature was an important predictor of PSSDs, which highlighted the potential of microbiota as a promising biomarker for detecting PSSD patients.

## 1. Introduction

Post-stroke sleep disorder (PSSD) is a common psychiatric complication following a stroke, which has serious impacts on the rehabilitation and life quality of stroke survivors [1]. PSSDs are highly prevalent, mainly occurring within 3–4 months after a stroke [2]. Growing data support that sleep disorders play a pivotal role as a risk factor and contribute to worsening stroke outcomes. PSSDs were found to be involved in many complex factors such as brain infarct regions, neurotransmitter disorder, sleep–wake system impairment, psychosocial factors, environmental factors, and so on [3]. Ischemia and hypoxia lead to the irreversible injury of neurons, which release a large number of toxic substances such as excitatory amino acids, hinder the conduction of the ascending reticular activation system, affect the sleep–wake mechanism, change the sleep rhythm, and lead to sleep disorder [4]. Sleep disorders could directly affect the stroke rehabilitation, quality of life, and neurological recovery of stroke survivors, and even increase the risk of stroke recurrence [5]. Currently, most patients with PSSDs are difficult to identify early, and it is very necessary to explore early biomarkers of PSSDs in the clinic.

Alterations in gut microbiota have been associated with many psychiatric disorders [6]. A clinical study showed that the level of *Bacteroides* decreased two days after admission in patients with acute ischemic stroke and transient ischemic attack [7]. Recent studies have shown that abnormal microbiota could affect the prognosis after a stroke [8,9]. Houlden et al. revealed that stroke could lead to alteration in cecal microbiota composition, with specific changes according to the severity of the injury [10]. Stroke could lead to gut microbiota dysbiosis, which in turn worsens stroke outcomes [11,12]. Benakis et al. reported that the regulation of gut microbiota could reduce ischemic brain injury in mice [13]. Smith et al. reported a positive correlation between gut microbiome diversity and richness and quality of sleep, as well as a negative correlation between microbiome diversity and sleep fragmentation, identifying certain phyla and taxa related to sleep health [14]. Chronic sleep-fragmentation-induced gut microbiota changes are characterized by the preferential growth in highly fermentative members of *Lachnospiraceae* and *Ruminococcaceae* and a decrease in *Lactobacillaceae* families [15]. These features of microbiota dysbiosis have been shown to contribute to stroke and sleep dysfunction, suggesting that the gut microbiome could be an important factor linking the PSSDs associated with both of these conditions; this in turn highlights the delicate interplay between the brain, intestines, and microbiome after this acute brain injury [16], suggesting the valuable diagnostic potential of microbiota-derived signatures. The changes in gut microbiota may be involved in the occurrence and development of PSSDs. Despite considerable progress in linking gut microbiota with PSSDs, their microbiota features remain largely unknown. The contribution of the gut microbiota to PSSDs has not been found to result in the identification of effective biomarkers and clinical interventions to improve stroke outcomes. Therefore, there is a need to elucidate the relationship between gut microbiota and PSSDs.

In this study, we aimed to explore the characteristics of the gut microbiota of patients with PSSDs. In addition, we further confirmed the correlations between alterations in microbiota and the clinical parameters and their potential as a biomarker for the detection of patients with PSSDs.

## 2. Methods

### 2.1. Patients

This was a prospective study on the associations between gut microbiota and sleep disorders after acute ischemic stroke. Patients with acute ischemic stroke were recruited from the Second Affiliated Hospital and Yuying Children’s Hospital of Wenzhou Medical University from September 2020 to June 2021. The inclusion criteria were as follows: patients diagnosed with ischemic stroke, aged ≥ 18; treatment without antibiotics or probiotics; and admission to hospital within 7 days after stroke onset. The exclusion criteria were as follows: clinical diagnosis of sleep disorders prior to admission; unable to complete the scale assessment due to severe dysarthria, cognitive impairment, or mental illness; patients had experienced serious social life events within three months, such as divorce or being widowed; history of systemic diseases such as cirrhosis, systemic lupus erythematosus, renal failure, gut disease, such as inflammatory bowel disease, Crohn’s disease and ulcerative colitis, and malignant intestinal tumor, restrictive diet, gastrointestinal surgery, recent infection, psychosis such as schizophrenia or bipolar disease, severe life-threatening illnesses, communication deficits, and pregnancy.

### 2.2. Clinical Data Collection

After recruitment, baseline data were extracted from case reports in the hospital in the Department of Neurology. The extracted data included blood test results, such as measurements of fasting or random glucose, HbA1c, lipids, creatinine, urea, and uric acid; physical measurements, such as blood pressure, height, and weight; and urine test results (i.e., creatinine, cells, and formed elements). The degree of neurological impairment was evaluated using the National Institute of Health Stroke Scale (NIHSS). A diagnosis of sleep disturbance was defined as a Pittsburgh Sleep Quality Index (PSQI) score of ≥5 at admission, based on the PSQI measure to evaluate overall sleep quality during the previous month [17].

### 2.3. Gut Microbiota Analysis

The stool samples were collected within 24 h of admission and immediately stored at −80 °C prior to microbial analysis. Microbial DNA was extracted from stool samples using the E.Z.N.A.^®^ soil DNA Kit (Omega Bio-Tek, Norcross, GA, USA) according to the manufacturer’s protocols. The resulting PCR products were extracted from a 2% agarose gel and further purified using the AxyPrep DNA Gel Extraction Kit (Ax·ygen Biosciences, Union City, CA, USA) and quantified using QuantiFluor™-ST (Agora, Promega, USA) according to the manufacturer’s protocol. Purified amplicons were pooled in equimolar amounts and paired-end sequenced (2 × 300) on an Illumina MiSeq platform (Illumina, San Diego, CA, USA) according to the standard protocols of Majorbio Bio-Pharm Technology Co. Ltd. China. Raw FASTQ files were quality-filtered by Trimmomatic and merged by FLASH. The taxonomy of each 16 s rRNA gene sequence was analyzed by the RDP Classifier algorithm against the database using a confidence threshold of 70%. The linear discriminant analysis (LDA) effect size (LEfSe) was applied to find significantly enriched taxa and their influence between the two groups using a nonparametric Kruskal–Wallis (KW) sum rank test, with thresholds of LDA score >2. Moreover, the significantly different genera were also identified based on the relative abundance of community by Wilcoxon rank sum tests. The predicted KEGG orthologues were summarized at hierarchy level 3, and the differential abundances by group were determined.

### 2.4. Follow-Up

Three months after ischemic stroke onset, all enrolled patients were evaluated with questionnaires, including the PSQI and modified Rankin scale (mRS). Self-reported sleep habits over a one-month time span were characterized using the PSQI [18]. The PSQI was a self-rated questionnaire intended to comprehensively assess sleep quality by nineteen individual items to generate seven “component” scores: subjective sleep quality, sleep latency, sleep duration, habitual sleep efficiency, sleep disturbances, use of sleeping medication, and daytime dysfunction. The sum of the scores of these seven components yields one global score, which has a range of 0–21; higher scores indicate worse sleep quality, and a score of >5 indicates poor sleep quality. The mRS was defined categorically with seven different grades: 0 indicates no symptoms, 5 indicates severe disability, and 6 indicates death [19]. According to the follow-up results three months after discharge, patients with post-stroke sleep disorders were defined as patients with PSQI scores (out) >5, classified as the PSSD group. The others with PSQI scores (out) ≤5 were grouped as PSNSD group.

### 2.5. Statistical Analysis

All quantitative data were analyzed using SPSS 25.0 (Armonk, NY: IBM Corp). According to the Kolmogorov–Smirnov normal test, clinical data with normal distribution were expressed as mean ± standard deviation and clinical data with non-normal distribution were described by median and quartile. All statistical tests were conducted as nondirectional with α = 0.05. Logistic regression was used to identify factors with a significant effect on the PSQI scores in PSSD subjects. The odds ratio (OR) and 95% confidence intervals (CIs) were determined; *p* < 0.05 showed that there was a significant difference between the groups.

## 3. Results

### 3.1. Patients’ Characteristics

The baseline information of the patients is shown in Table 1. Among the 205 patients, 74 met the diagnostic criteria of PSSD. Most of the patients were elderly, with 67.8% >60 years old. There were no significant differences in sex, diabetes mellitus, hypertension, current smoking, and alcohol status between the PSSD and PSNSD groups (*p* > 0.05). However, there were significant differences in terms of the TG, TSH, and PSQI scores between the two groups (*p* < 0.001 and *p* < 0.001, respectively). The PSSD group showed significantly higher values in the TG and TSH than those in the PSNSD group.

### 3.2. Multivariable Logistic Regression Analysis of the Risk Factors for PSSD

As shown in Table 2, the assessment of factors predicting PSSDs was carried out by multivariable logistic regression analysis. PSSDs were independently associated with a higher baseline NIHSS score (OR 1.388, 95% CI 1.024–1.882, *p* = 0.034) and higher TSH level (OR 1.363, 95% CI 1.045–1.778, *p* = 0.022).

### 3.3. Changes in Microbiota Diversity and Community Type in Patients with PSSD

**Figure 1:**
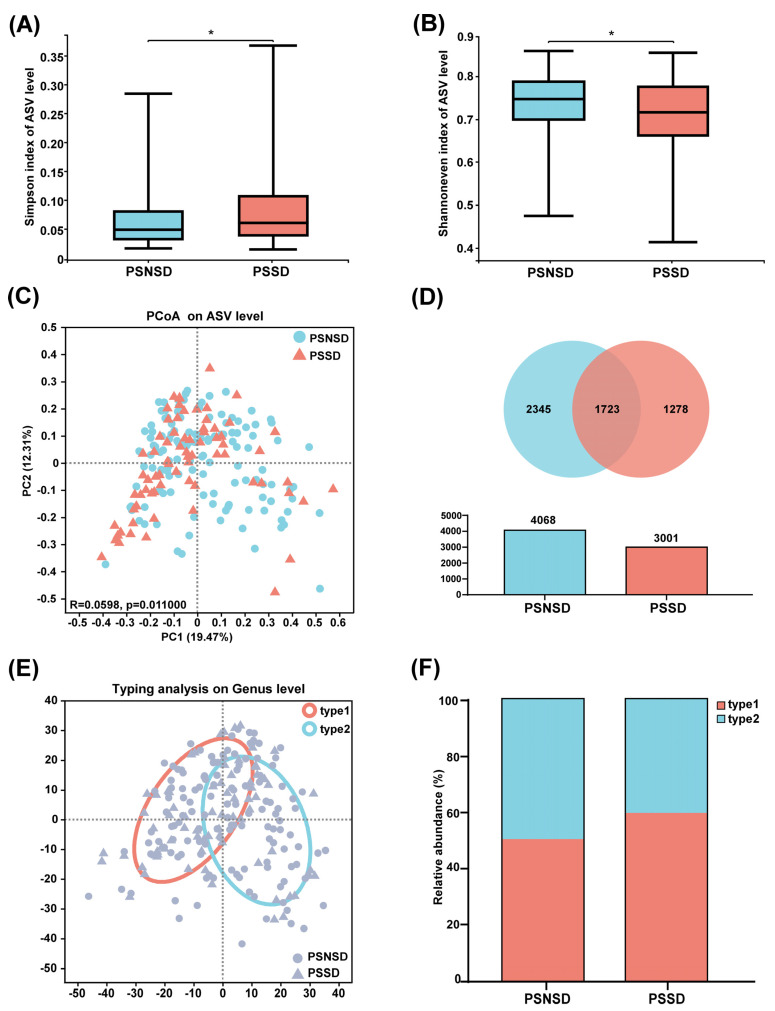
Changes in microbiota diversity and community type in patients with PSSD. (**A**) Simpson and (**B**) Shannon even indexes. (**C**) Principal coordinate analysis (PCoA) plots. (**D**) Venn diagram of fecal microbiota at the ASV level. (**E**) The circle colored by enterotype: orange-red color corresponds to enterotype 1 (*Escherichia-Shigella*) and blue color corresponds to enterotype 2 (*Bacteroides*). (**F**) The percentages of enterotype 1 and enterotype 2 in the PSSD and PSNSD samples. *: *p* < 0.05.

The full dataset included bacteria from 73 genera, 37 families, 14 orders, 8 classes, and 5 phyla. Gut microbiota in the PSSD group showed significantly lower α-diversity compared with the PSNSD group (Figure 1A). Although no statistically significant differences were found with respect to commonly used richness indices (Sobs, Chao1, and ACE, all *p* > 0.05), comparisons of the Shannon and Simpson indices of the PSSD and PSNSD samples showed a significant difference in the evenness of gut microbiota (Figure 1B). Furthermore, the PCoA scatterplot of fecal microbiota was significantly different between the PSSD and PSNSD groups (Figure 1C). We evaluated β-diversity based on the weighted (quantitative, ANOSIM statistic = 0.0598, *p* = 0.011) UniFrac distance matrix of the differences between groups. The Venn diagram (Figure 1D) shows the logical relationships between the ASV numbers of the two sets. The number of shared ASVs was 1723. The number of unique ASVs was 1278 in the PSSD group and 2345 in the PSNSD group. Thus, the number of unique ASVs in the feces of the PSNSD group is higher than in the PSSD group. The 205 samples were divided into two clusters (Figure 1E). Enterotype 1 was dominated by *Escherichia-Shigella* as the most enriched genus, and *Bacteroides* was the core in enterotype 2. Interestingly, there was a dysbiosis of enterotype distribution by PSSD conditions. For the PSNSD group, the percentage of samples in both enterotypes was the same (50% in enterotype 1, 50% in enterotype 2), whereas a higher percentage of patients with PSSDs were found to be distributed in enterotype 1 (60%) compared to enterotype 2 (Figure 1F). Therefore, a morbid state of PSSD was associated with imbalanced microbial communities, with a tendency toward the enterotype dominated by *Escherichia-Shigella* and away from the *Bacteroides* enterotype.

### 3.4. Changes in the Microbial Composition of PSSD Patients

**Figure 2:**
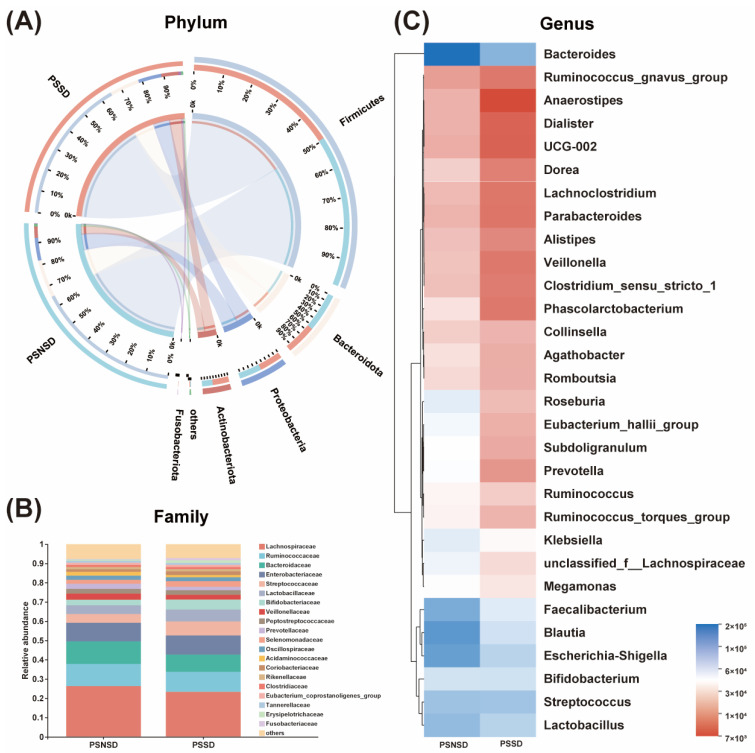
Alterations in the composition of gut microbiota in patients with PSSD. (**A**) Distribution of the microbial community for each group at the phylum level. (**B**) Average relative abundances of microbial community composition for each group are shown by bar plots for the family level. (**C**) Heatmap of gut microbiota composition at the genus level for the PSSD and PSNSD groups. PSSD: Post-stroke sleep disorder; PSNSD: Non-sleep disorders after stroke.

**Figure 3:**
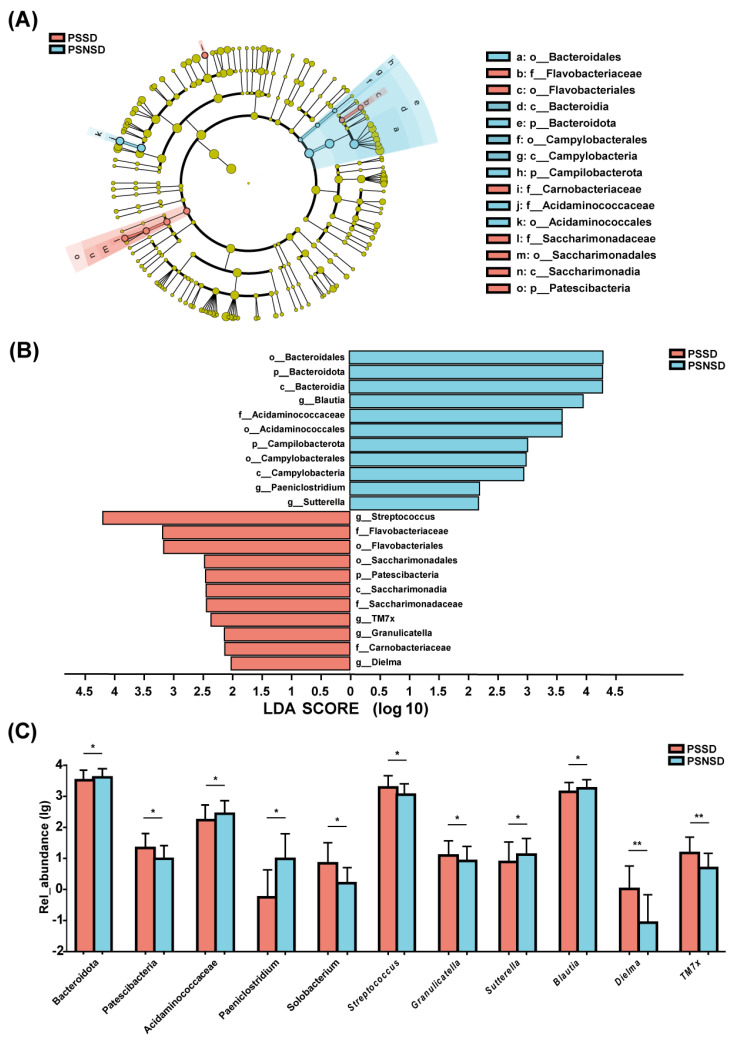
LEfSe analysis of taxonomic biomarkers of gut microbiota. Cladogram showing the phylogenetic relationships of the bacterial taxa and Linear discriminant analysis (LDA) scores between the PSSD and the PSNSD groups (**A**,**B**). The relative abundance of gut microbiota at the phylum, family, and genus level. (**C**) Differences were assessed by rank sum test and denoted as follows: the relative abundances of the significant bacteria at the phylum level, the Familyand the genus level in PSSD patients compared with PSNSD patients. * *p* < 0.05 and ** *p* < 0.01. PSSD: Post-stroke sleep disorder; PSNSD: Non-sleep disorders after stroke.

The overall gut microbial compositions are shown in Figure 2 at the phylum, family, and genus levels, respectively. As shown in Figure 3, a total of three phyla, one family, and the top eight genera with significant differences were identified and are shown among the two groups. At the phylum level, *Firmicutes*, *Bacteroidetes*, and *Proteobacteria*, followed by *Actinobacteria* were the predominant phyla in each group with relative abundances of >80% (Figure 2A). At the family level, the gut microbial population is dominated by *Lachnospiraceae*, *Ruminococcaceae*, *Bacteroidaceae*, *Enterobacteriaceae*, *Streptococcaceae*, *Lactobacillaceae*, *Bifidobacteriaceae*, *Veillonellaceae*, *Peptostreptococcaceae*, and *Prevotellaceae* (Figure 2B). The bacterial taxonomy distribution and heatmap of the PSSD group showed decreased density and clustering compared to the PSNSD group (Figure 2C). At the genus level, lower abundances were observed for *Bacteroides*, *Blautia*, *Bifidobacterium*, *Lactobacillus*, *Faecalibacterium*, and *Klebsiella* in the PSSD group compared with the PSNSD group.

### 3.5. LEfSe Analysis of the Taxonomic Biomarkers of Gut Microbiota

As shown in Figure 3, there were significant differences in the composition of gut microbiota between PSSD and PSNSD groups at the phylum, family, and genus levels. The LEfSe algorithm approach was applied (LDA score >2.0). The PSSD group showed the most unique microbiota, with a high abundance of *Streptococcus*. The abundance of genus *Blautia* was higher in the PSNSD group (Figure 3A). There were three taxa at the phylum level and four taxa at the family level (Figure 3B). At the phylum level, patients with PSSDs had a significantly higher content of *Patescibacteria*, and the PSNSD group had significantly higher contents of *Bacteroidota* and *Campilobacterota* (Figure 3C). At the family level, the PSNSD group had a higher content of *Acidaminococcaceae* (Figure 3C). At the genus level (Figure 3C), PSSD patients had significantly higher levels of *Streptococcus*, *TM7x*, *Granulicatella*, and *Dielma*; and lower contents of *Blautia*, *Paeniclostridium*, and *Sutterella* (Figure 3C).

### 3.6. Correlations of Gut Microbes and Clinical Characteristics

**Figure 4:**
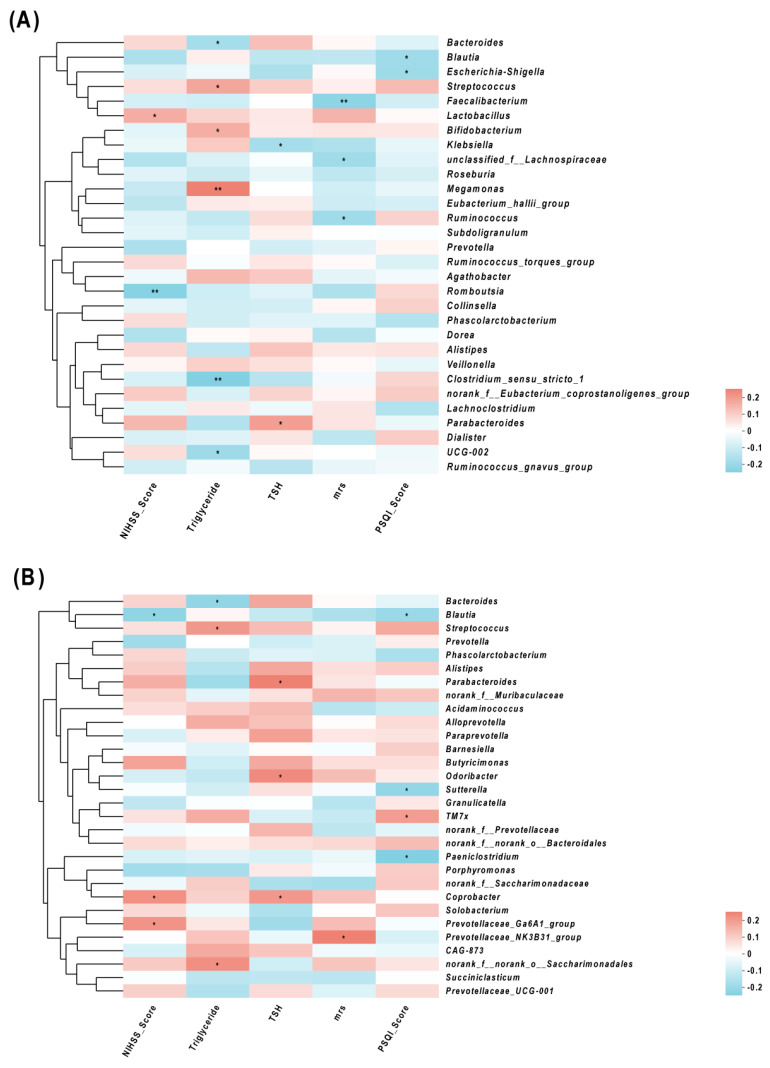
Correlations of the relative abundance of gut microbiota and clinical characteristics, (**A**) and (**B**) between clinical parameters and gut microbiota were analyzed using Spearman’s analysis (heatmap). The *x*-axis represents the clinical parameters. The *y*-axis represents gut microbiota in the top 30 in terms of abundance (**A**) and the gut microbiota with differential abundance (**B**). The colors of the grids represent the correlation analysis value of Spearman’s correlation analysis. Grids in red indicate positive correlations (correlation analysis value more than 0.1), while grids in blue indicate negative correlations (correlation analysis value less than −0.1). The color-coded scale indicates the correlation analysis value from the heatmap; deeper red or blue indicates higher correlation values. * *p* < 0.05 and ** *p* < 0.01.

Spearman correlation analyses showed that the abundances of *Streptococcus* and *Megamonas* were positively correlated with TG, and the abundance of *Bacteroides* was negatively correlated with TG. The abundances of *Blautia* and *Romboutsia* were negatively correlated with NIHSS, while the abundances of *Lactobacillus* and *Coprobacter* were positively correlated with NIHSS. The abundances of *Parabacteroides* and *Coprobacterium* were positively correlated with TSH, while the abundance of *Klebsiella* was negatively correlated with TSH. An elevated PSQI score was also associated with a reduction in *Blautia*, *Sutterella*, *Paeniclostridium*, and *Escherichia-Shigella*. Furthermore, a positive relationship was observed in the PSQI with the abundance of *TM7x* (Figure 4).

### 3.7. Predictor Performance of Gut Microbiota

**Figure 5:**
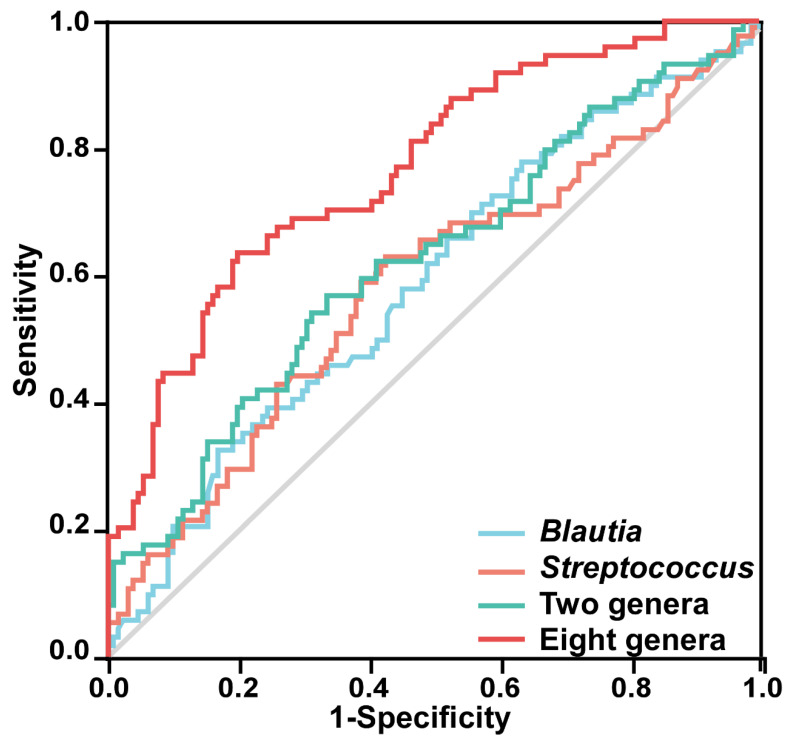
ROC curves are demonstrated based on the relative abundance of the eight characteristic genera in discriminating PSSDs from PSNSDs. Each curve in the figure represents the ROC curve of the best model using different microbiota combinations: *Blautia* alone (AUC = 0.590, 95% CI: 0.509–0.671, *p* = 0.032), *Streptococcus* alone (AUC = 0.588, 95% CI: 0.505–0.671, *p* = 0.036), combination of two genera (*Blautia* and *Streptococcus*), and combination of eight genera (*Streptococcus*, *TM7x*, *Granulicatella*, *Dielma*, *Blautia*, *Paeniclostridium*, *Sutterella*, and *Blautia*).

The predictive model based on *Streptococcus* and *Blautia* distinguishes PSSD patients from non-PSNSD patients (AUC = 0.620, 95% CI: 0.539–0.702, *p* = 0.004, Figure 5). Then, the combination of the gut taxa further improved the discriminative power. Compared with the single taxa, the combination of the eight gut taxa showed relatively higher classification accuracy, with an AUC of 0.768 (95% CI: 0.701–0.835, *p* < 0.001, Figure 5).

### 3.8. Gut Microbial Function

**Figure 6:**
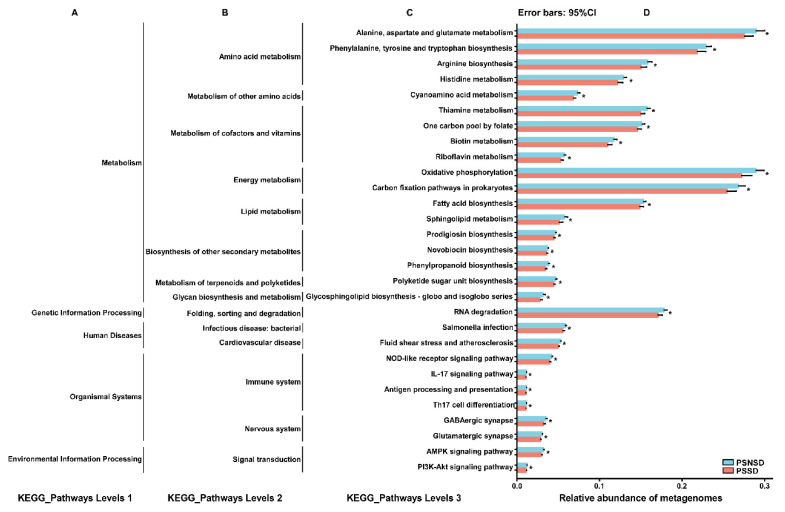
Relative abundance of the KEGG pathways of functional categories in the gut microbiota between the PSSD group and the PSNSD group. (**A**). KEGG_Pathways Levels 1; (**B**). KEGG_Pathways Levels 2; (**C**). KEGG_Pathways Levels 3; (**D**). Relative abundance of metagenomes. Significant differences in KEGG pathways at level 3 were detected using SPSS software based on the KEGG pathway database. * *p*  <  0.05.

The 16 s rRNA sequencing data were categorized into 328 KEGG functional pathways; the pathways present in <10% of participants were removed, leaving 284 KEGG pathways for comparison. A total of 29 pathways at KEGG level 3, including cardiovascular disease, nervous system, and immune system, were significantly different between the two groups. Compared with the PSNSD group, the GABAergic synapse pathway, glutamatergic synapse pathway, and amino acid metabolism pathway were significantly downregulated in the PSSD group (Figure 6).

## 4. Discussion

This study showed that SCFA-producing bacteria were low in patients with PSSD. Moreover, *Blautia, Sutterella*, *Paeniclostridium*, and *Escherichia-Shigella* were also negatively correlated with PSQI scores. Furthermore, the ROC curve models based on characteristic *Streptococcus* and *Blautia* distinguished PSSD patients from non-PSNSD patients, and the combination of the eight gut taxa showed relatively higher classification accuracy. These results indicated that gut microbiota might be a novel biomarker for PSSD.

In this study, there were 3 candidate taxa associated with gut microbial dysbiosis at the phylum level, 4 at the family level, and 10 at the genus level in the PSSD group. The patients with PSSDs had a higher abundance of *Firmicutes*, a lower abundance of *Bacteroidetes*, and an increased ratio of *Firmicutes*/*Bacteroidetes* (F/B). An increased F/B ratio has been associated with numerous nervous system diseases, such as stroke and sleep deprivation [15,20,21]. An increase in the F/B ratio has also been linked with increased mortality after middle cerebral artery occlusion (MCAO), reduced functional prognosis, and increased levels of systemic proinflammatory cytokines [22], which are involved in the progression of PSSD. The patients with PSSDs had a lower abundance of *Blautia,* which is a kind of SCFA-producing bacteria, compared with patients with PSNSD.

In this study, the richness within the *Blautia, Sutterella*, *Paeniclostridium*, and *Escherichia-Shigella* was negatively correlated, and the abundance of the *Bacteroides* phylum was positively correlated with sleep quality, which was consistent with previous studies [14,23]. We observed the associations of TG, TSH, and NIHSS with gut microbiota. A recent study showed that a combination of pro- and pre-biotics could significantly reduce TSH [24]. The change in gut microbes is significantly correlated with the hormone level of the hypothalamic–pituitary–thyroid axis [25]. Recent studies have shown that the level of TSH was elevated in different types of patients with sleep disorders and TSH values were positively correlated with nocturnal sleep deprivation [26,27,28]. A clinical trial showed that the proportions of poor sleep and occasional poor sleep in subjects with isolated TSH elevation were significantly higher than that in subjects with normal TSH levels, which shows that TSH affects the quantity and architecture of sleep [29,30]. As the secretion of TSH is controlled by circadian rhythms, poor sleep is often accompanied by an increase in the TSH level [29]. TSH secretion exhibited clear daily rhythmicity, and chronic sleep debt disrupts rhythmic TSH secretion [31]. Low TSH values were positive for maintaining slow wave sleep and normal sleep structure, while the hyper-secretion of active TSH adversely affects the quality and quantity of sleep [30]. When the sleep status improved, the isolated elevated TSH concentration returned to normal [32]. The beneficial microbial metabolite SCFAs could maintain HPT axis stability [33]. Improving plasma triglyceride levels by enriching SCFA producers such as *Blautia* could alleviate hyperlipidemia in diabetes [34]. Decreased sleep quality was independently associated with increased levels of triglycerides [35]. Wrzosek et al. reported that significant correlations were found between the PSQI scores and serum triglycerides [36]. Compared with normal sleep time, insomnia in patients with sleep disorders was often accompanied by an increase in serum triglycerides [37]. Insomnia was related to most cases of cardiovascular and cerebrovascular diseases, some of which may be partially mediated by high triglycerides [38]. Recently it was also shown that microbiota were substantial drivers of circulating lipid levels, including triglycerides [39]. A potential part of the biological basis for the association between circulating lipid levels and microbial taxa may be through SCFAs. Several taxa (*Lachnospiraceae*, *Corynebacterium*, and *Blautia*) were negatively correlated with sleep measures. The abundances of several inflammation-related strains (*Proteobacteria*, *Clostridiaceae*, *Oscillospiraceae*, and *Klebsiella*) were found significantly modified in relation to sleep parameters [40]. The NIHSS was devised to measure stroke severity and has been a widely used outcome measure after a stroke [41]. The higher NIHSS score in the PSSD group seemed to show the influence of PSSDs on the increase in adverse prognoses in patients with ischemic stroke. Importantly, ROC curves demonstrated that the eight characteristic gut microbiota had the potential to distinguish PSSDs from PSNSDs, suggesting that gut microbiota might serve as a minimally invasive and cost-effective index for screening PSSD.

In this study, KEGG revealed that the altered metabolism induced by gut microbiota, such as amino acid metabolism, metabolism of cofactors and vitamins, and lipid metabolism, may contribute to sleep disorders in patients with ischemic stroke. In addition, sleep disorders could produce alterations in cardiovascular disease, and the immune system, such as fluid shear stress and atherosclerosis, NOD-like receptor signaling pathway, and IL-17 signaling pathway, induced by gut microbiota. GABA, as the main inhibitory neurotransmitter in the brain, has been shown to have an effect on stress and sleep [42]. Currently available evidence suggests that gut microbes might play a role in regulating GABAergic signals, thereby exerting some hypnotic and antianxiety effects [43,44]. During non-REM sleep up/down-state transitions, the up-states emerge from coordinated signaling between glutamatergic and GABAergic synapses [45]. Gut microbiota could influence the coordinated activity of inhibitory GABAergic interneurons with excitatory glutamatergic pyramidal cells [46]. Taken together, it is likely that these KEGG pathways provide a new functional view for understanding the gut microbiota that contribute to PSSDs.

Our results indicated that gut microbiota composition might be an important influencing factor for PSSD. Further studies are needed to compare the predictive value of gut microbiota with that of other markers and to explore these microbial parameters in the context of different types of ischemic stroke neutralization therapy. It was also critical to pay attention to the dynamic changes in gut microbiota over time in PSSD patients to identify potential favorable versus hostile microbiota. Clinical research shows that probiotics had good prevention and treatment effects on autism spectrum disorder, Alzheimer’s disease, Parkinson’s disease, depression, multiple sclerosis, and other nervous system diseases, and were also being developed in other nervous system diseases [47,48]. Therefore, the determination of baseline parameters helped to determine the risk of serious adverse events in patients with PSSDs.

Several limitations of this study should be noted. First, central sleep apnea is a high possibility after ischemic stroke, and we failed to detect central sleep apnea by polysomnography, which restricted the investigations of different PSSD types. Second, arrhythmia such as atrial fibrillation is a strong risk factor for ischemic stroke, and it might have an impact on the results. Third, we only collected fecal samples once and did not observe dynamic changes in gut microbiota after the stroke onset. Nevertheless, the results of this study still provided insights into the predictive role of gut microbiota in the prognosis of post-stroke sleep disorders which was not reported before.

To conclude, PSSD patients had an altered gut microbiota composition, which was closely related to the clinical parameters. The characteristic gut microbiota might facilitate the diagnosis of PSSDs, which might open new avenues for the targeted prevention and treatment of PSSDs.

## Figures and Tables

**Table 1:** Demographic and clinical characteristics of PSSD subjects versus PSNSD subjects.

	PSSD (*n* = 74)	PSNSD (*n* = 131)	*p*-Value
Socio-demographic factors			
Male subjects, *n* (%)	46 (62.2)	92 (70.2)	0.248
Age (years, mean ± SD)	66 ± 12	65 ± 12	0.613
Marital status			0.876
Married	65 (87.8)	114 (87.0)	
Unmarried	1 (1.4)	2 (1.5)	
Divorced	8 (10.8)	15 (11.5)	
Education			0.276
Illiterate	14 (18.9)	24 (18.3)	
Elementary school	33 (44.6)	49 (37.4)	
Middle school	20 (27)	41 (31.3)	
High school and above	7 (9.5)	17 (12.9)	
Health-related behaviors			
Smoking, formerly/currently	27 (36.5)	51 (38.9)	0.731
Drinking, formerly/currently	28 (37.8)	49 (37.4)	0.951
History of disease			
Atrial fibrillation	7 (9.4)	10 (7.6)	0.848
Hypertension	51 (68.9)	93 (71)	0.757
Diabetes mellitus	28 (37.8)	45 (34.4)	0.619
CVD history	12 (16.2)	21 (16.0)	0.972
Hyperlipemia	32 (43.2)	53 (40.5)	0.699
Clinical parameters			
MAP (mmHg)	108.37 ± 14.14	110.22 ± 13.51	0.354
ALT (u/L)	18 (13–25.25)	17 (12–24)	0.327
AST (u/L)	19.5 (16–23)	18 (15–22)	0.215
Creatinine (μmol/L)	63.2 (56.23–73.6)	62.9 (51.9–76.5)	0.992
Folate (nmol/L)	9.29 (6.11–11.49)	9.61 (7.21–13.47)	0.191
B12 (pg/mL)	332 (235.5–406)	355 (234–515)	0.327
UA (μmol/L)	296 (237.25–356.75)	314 (246–364)	0.400
Hcy (μmol/L)	10.7 (9.3–13.1)	11.1 (9.1–13.7)	0.582
hs-CRP (mg/L)	1.675 (0.78–4.72)	1.23 (0.61–3.42)	0.271
TG (mmol/L)	1.66 (1.28–2.1)	1.45 (1.08–1.78)	0.003 *
TC (mmol/L)	4.46 ± 1.04	4.37 ± 1.13	0.590
LDL (mmol/L)	2.87 ± 0.95	2.84 ± 1.07	0.880
HDL (mmol/L)	1.07 (0.84–1.27)	1 (0.84–1.23)	0.348
FPG (mmol/L)	5.41 (4.93–6.71)	5.49 (4.85–6.33)	0.650
HbA1C (%)	6.08 (5.62–7.00)	5.9 (5.46–7.19)	0.618
FT3 (pg/mL)	2.85 (2.64–3.13)	2.92 (2.67–3.21)	0.234
FT4 (ng/dL)	1.13 (1.13–1,13)	1.14 (1.04–1.27)	0.224
TSH (μIU)	2.58 (2.56–2.58)	1.66 (1.02–2.66)	<0.001 *
NHISS in	2 (1–4)	2 (1–3)	0.250
PSQI in	2 (2–3)	2 (1–3)	0.480
PSQI out	8 (7–10)	3 (2–4)	<0.001 *
mRS out	1 (1–2)	1 (0–2)	0.194

Abbreviations: PSSD, Post-stroke sleep disorder; PSNSD, Non-sleep disorders after stroke; MAP, mean arterial pressure; CVD, cardiovascular disease; FPG, fasting plasma glucose; HDL-cholesterol, high-density lipoprotein cholesterol; AST, aspartate aminotransferase; ALT, alanine aminotransferase; hs-CRP, high-sensitivity C-reactive protein; HbA1c, hemoglobin A1c; B12, vitamin B12; Hcy, homocysteine; LDL-cholesterol, low-density lipoprotein cholesterol; FT3, free triiodothyronine; FT4, free tetraiodothyronine; TSH, thyroid-stimulating hormone; in, recorded at admission; out, recorded at three months after the stroke. * *p* < 0.05

**Table 2:** Multivariable logistic regression model predicting PSSD.

Variable	Multivariate			
	B (SE)	OR	95% CI	*p*
TG (mmol/L)	0.391 (0.205)	1.478	0.989–2.208	0.057
TSH (μIU)	0.243 (0.111)	1.275	1.026–1.585	0.028 *
NHISS	0.139 (0.062)	1.149	1.017–1.298	0.026 *

TSH, thyroid-stimulating hormone; * *p* < 0.05.

## Data Availability

Available upon request.
